# Transcranial direct current stimulation as a treatment for auditory hallucinations

**DOI:** 10.3389/fpsyg.2015.00244

**Published:** 2015-03-06

**Authors:** Sanne Koops, Hilde van den Brink, Iris E. C. Sommer

**Affiliations:** Psychiatry Department, Brain Center Rudolf Magnus, University Medical Center UtrechtUtrecht, Netherlands

**Keywords:** auditory hallucinations, focal stimulation, tDCS, psychosis, voices

## Abstract

Auditory hallucinations (AH) are a symptom of several psychiatric disorders, such as schizophrenia. In a significant minority of patients, AH are resistant to antipsychotic medication. Alternative treatment options for this medication resistant group are scarce and most of them focus on coping with the hallucinations. Finding an alternative treatment that can diminish AH is of great importance. Transcranial direct current stimulation (tDCS) is a safe and non-invasive technique that is able to directly influence cortical excitability through the application of very low electric currents. A 1–2 mA direct current is applied between two surface electrodes, one serving as the anode and the other as the cathode. Cortical excitability is increased in the vicinity of the anode and reduced near the cathode. The technique, which has only a few transient side effects and is cheap and portable, is increasingly explored as a treatment for neurological and psychiatric symptoms. It has shown efficacy on symptoms of depression, bipolar disorder, schizophrenia, Alzheimer’s disease, Parkinson’s disease, epilepsy, and stroke. However, the application of tDCS as a treatment for AH is relatively new. This article provides an overview of the current knowledge in this field and guidelines for future research.

## Hallucinations in Psychotic Disorders

Auditory hallucinations (AH) are a frequent symptom of schizophrenia which occurs in about 60–80% of patients (Andreasen and Flaum, 1991). The content of these hallucinations is mostly negative, often conveying anger (McCarthy-Jones et al., 2014), or terms of abuse (Nayani and David, 1996) and increasing the risk for suicidal behavior (Harkavy-Friedman et al., 2003). Furthermore, AH also occur in other diagnostic groups such as borderline personality disorder, anxiety disorders, affective disorders, posttraumatic stress disorder, autism, and significant hearing loss (Sommer et al., 2012b).

In approximately 70–75% of these cases, antipsychotic medication and/or cognitive behavioral therapy sufficiently suppress AH. However, the remaining 25–30% continues to suffer from AH despite optimal therapy (Shergill et al., 1998). Alternative treatment options are scarce for this group of patients, and often focus on coping with the hallucinations and accepting their presence instead of reducing them (Bentall et al., 1994). Due to the distressing impact AH have on the patients’ quality of life, their ability to concentrate and their social and professional functioning, finding an effective treatment option to reduce AH in this treatment-resistant group would be of great value.

## Transcranial Direct Current Stimulation

Transcranial direct current stimulation (tDCS) is a non-invasive focal neurostimulation technique that involves the application of a low intensity electric current between two surface electrodes, an anode and a cathode (Nitsche et al., 2008). A 1–2 mA direct current is applied between two electrodes, often with a size of 35 cm^2^ (5 cm × 7 cm), which are placed on the scalp. The current flows between the anode and the cathode, some of it being diverted through the scalp and another part moving through the brain where brain activity is modulated. Although the exact mechanisms of action of tDCS have yet to be completely elucidated, there are two mechanisms by which tDCS modulates brain activity that are currently widely accepted in the field (Brunoni et al., 2012). The first proposes that the current that passes through the brain modulates levels of cortical excitability in a polarity dependent manner (Miranda et al., 2006). Cortical excitability is thought to be increased in the vicinity of the anode and reduced near the cathode. The anodal and cathodal effects of tDCS on cortical excitability are thought to be explained by shifts in the resting membrane potential (depolarization and hyperpolarization). The second proposes that prolonged effects of tDCS can be explained by modifications of *N*-methyl-d-aspartate (NMDA) receptor efficacy, changes in gamma-aminobutyric acid-ergic activity and modulation of interneurons, resulting in prolonged synaptic efficacy changes (Nitsche et al., 2008; Stagg and Nitsche, 2011). However, most of what we know about the effects of tDCS on a neural level is derived from studies of the primary motor cortex (Nitsche et al., 2008). Questions can be raised concerning the generalizability of these underlying mechanisms to other cortical areas (Horvath et al., 2015).

In the 1960s, tDCS was studied extensively, but never gained much popularity in clinical practice (Priori, 2003). Currently, this seems to be changing as tDCS is increasingly explored for the treatment of both neurologic and psychiatric symptoms (Boggio et al., 2008). It has shown efficacy on the clinical symptoms of depression, on both depressive and manic symptoms of bipolar disorder, cognitive functioning in schizophrenia, episodic memory in Alzheimer’s disease, motor symptoms and working memory in Parkinson’s disease, on the amount of insults in epilepsy, on rehabilitation of motor function, and language disorder after stroke and on tinnitus (Floel, 2014; Kuo et al., 2014). tDCS is a safe treatment with few, if any, side effects (Nitsche et al., 2008). Some mild side effects of a transient nature that have been mentioned in association with tDCS are a slight headache, an itching or tingling sensation at the location of the electrodes and redness of the skin at the location of the electrodes. However, such side effects are also reported after placebo (sham) treatment (Brunoni et al., 2014). Other advantages of this technique are that tDCS equipment is relatively cheap, the treatment can be given after a short training course, and it is easily portable.

Compared to transcranial magnetic stimulation (TMS), another form of focal stimulation that has often been used as an alternative treatment option for AH (Slotema et al., 2014), tDCS has some explicit benefits when using it for research. It is easier to apply an identical placebo treatment, as less physical sensations occur compared to TMS. The itching or tingling sensation that is sometimes mentioned by participants is often only present at the start of treatment and disappears quickly. To mimic this in a placebo treatment, it helps to start with a short period of active stimulation (30–40 s) after which stimulation slowly regresses. During the rest of the treatment period, no active stimulation takes place. This short active stimulation period does not have modulating effects on the brain, but it properly mimics the physical sensations of active treatment. Furthermore, it is possible to preprogram tDCS equipment to apply placebo treatment, which makes it easier to perform double blind sham-controlled trials (Nitsche et al., 2008).

## Transcranial Direct Current Stimulation in AH

The use of tDCS as a treatment option for medication resistant AH is relatively new and has till now only been tested in patients with AH in the context of psychotic disorders. Homan et al. (2011) were the first to explore this in a case study. For 10 consecutive days, the patient received daily treatment of 15 min tDCS at an intensity of 1 mA. The cathode was placed on the left temporoparietal cortex (TPC), a location that has been linked to the experience of AH (Silbersweig et al., 1995; Shergill et al., 2000). The anode was placed on the right supraorbital area. Using this method, they were able to ameliorate medication resistant AH. Moreover, this effect was still present 6 weeks after treatment. A more recent case study reported a reduction of 90% in AH severity after 2 months of daily 20 min tDCS at an intensity of 1 mA (Andrade, 2013). After this, treatment was increased to twice daily and an intensity of 3 mA, which led to increased improvement in AH severity. This improvement was still present after 3 years of daily maintenance treatment, but when treatment frequency was reduced to once per 2 days the ameliorating effects disappeared. In this study, the cathode was also placed on the left TPC, but the anode was placed on the left dorsolateral prefrontal cortex (DLPFC). This is an area that is often used in studies investigating the effects of tDCS on cognitive functioning (Hoy et al., 2014). These two tDCS locations have been used in several other case studies reporting positive effects of tDCS on AH severity after multiple sessions of 20 min tDCS at an intensity of 2 mA (Rakesh et al., 2013; Shiozawa et al., 2013; Shivakumar et al., 2013; Nawani et al., 2014). However, all these studies were open-label without a control condition, meaning that non-specific effects (i.e., placebo-effects) may have been of large influence. Brunelin et al. (2012) were the first to publish a tDCS study in AH in which a control group was included. In this study, 15 patients received a 20 min tDCS treatment twice daily at an intensity of 2 mA, for 5 consecutive days and an equal number of patients received sham. In this study, the cathode was also placed on the left TPC and the anode on the left DLPFC. They found a robust decrease in AH severity of 30% compared to a placebo group of equal size. Furthermore, a significant decrease was found in other symptoms as measured by the Positive and Negative Syndrome Scale (PANSS), such as negative symptoms. These positive effects were still present after 3 months. A more recent study by the same group, using a partially overlapping sample of 28 patients and identical methods, reported a decrease of 46% in AH frequency in medication resistant patients after active treatment, whereas no such effect occurred in the placebo group (Mondino et al., 2014). A recent open label study including 21 patients with schizophrenia and the same stimulation parameters and locations as Brunelin et al. (2012) found a similar improvement of 32% in AH severity. However, this study did not include a placebo condition (Bose et al., 2014). Up till now, one pilot study has been published that reports no beneficial effect of tDCS treatment on AH (Fitzgerald et al., 2014). This study tested the effects of both unilateral (*N* = 13) and bilateral (*N* = 11) tDCS on AH compared to placebo. In total, patients underwent 15 treatments in 3 weeks time. The AH did not respond to either treatment method. However, it is important to note that this study had a very small sample size and rated AH severity based on the PANSS only. This scale does not measure AH in much detail and the different severity scores of the hallucination item are found to be hard to differentiate (Santor et al., 2007). Other studies mostly use the Auditory Hallucination Rating Scale (AHRS) to measure AH severity. See **Table 1** for a summary of all includedstudies.

**Table 1:** Studies using tDCS for the treatment of auditory hallucinations (AH).

Reference	Design	Patients	Position of electrodes	Current strength	Duration	Effects
Andrade (2013)	Case study	One subject with schizophrenia and clozapine resistant AH.	The cathode over the left temporoparietal cortex (TPC) and the anode over the left dorsolateral prefrontal cortex (DLPFC).	First 1 mA, later 3 mA	First 20 min (daily sessions for 2 consecutive months), later 30 min (twice daily sessions for 3 consecutive years).	A reduction of 90% in AH severity was reported after the first 2 months. After increasing treatment to twice daily and a current intensity of 3 mA, AH severity further improved. When electrode positioning was changed or treatment frequency was reduced to once per 2 days, the ameliorating effects disappeared.
Bose et al. (2014)	Open label study, without a placebo condition	21 subjects with schizophrenia and medication resistant AH.	The cathode over the left TPC and the anode over the left DLPFC.	2 mA	20 min (twice daily sessions on 5 consecutive days).	Improvement of 32% in AH severity.
Brunelin et al. (2012)	Randomized, placebo controlled, double-blind trial	30 subjects with schizophrenia and medication resistant AH.	The cathode over the left TPC and the anode over the left DLPFC.	2 mA	20 min (twice daily sessions on 5 consecutive days).	Improvement of 31% in AH severity which lasted for 3 months.
Fitzgerald et al. (2014)	Randomized, placebo controlled, double-blind trial	24 subjects with schizophrenia or schizoaffective disorder and medication resistant AH.	Unilateral and bilateral tDCS with the cathode over the (left) TPC and the anode over the (left) DLPFC.	2 mA	20 min (daily sessions on 15 days in 3 consecutive weeks)	AH did not respond to either treatment method, and no difference was found between the active and placebo treatment group.
Homan et al. (2011)	Case study	One subject with schizophrenia and medication resistant AH.	The cathode over the left TPC and the anode over the right supraorbital area.	1 mA	15 min (daily sessions on 10 consecutive days)	Amelioration of AH was reported, which was still present 6 weeks after treatment.
Mondino et al. (2014)	Randomized, placebo controlled, double-blind trial	28 subjects with schizophrenia and medication resistant AH.	The cathode over the left TPC and the anode over the left DLPFC.	2 mA	20 min (twice daily sessions on 5 consecutive days).	Improvement of 46% in AH severity in the active treatment group, with no such effect in the placebo group.
Nawani et al. (2014)	Case study	One subject with schizophrenia and medication resistant AH.	The cathode over the left TPC and the anode over the left DLPFC.	2 mA	20 min (twice daily sessions on 5 consecutive days).	Improvement of AH.
Rakesh et al. (2013)	Case study	One subject with schizophrenia and medication resistant AH.	The cathode over the left TPC and the anode over the left DLPFC.	2 mA	20 min (twice daily sessions on 5 consecutive days).	Complete cessation of AH.
Shiozawa et al. (2013)	Case study	One subject with schizophrenia and medication resistant AH.	The cathode over the left TPC and the anode over the left DLPFC.	2 mA	20 min (twice daily sessions on 5 consecutive days).	Improvement of AH, which was sustained after a 2-month follow-up.
Shivakumar et al. (2013)	Case study	One subject with schizophrenia and medication resistant AH.	The cathode over the left TPC and the anode over the left DLPFC.	2 mA	20 min (twice daily sessions on 5 consecutive days).	Complete cessation of AH, which was sustained after a 4-week follow-up.

It is still unclear in what way tDCS might influence AH. As all studies that report positive results up till now have used the left TPC as the location for the cathode, it appears that the inhibiting action of tDCS on that specific location is important for the beneficial effects. It is unclear if excitation on the left DLPFC also influences AH severity. Brunelin et al. (2012) associated excitation on this location with a decrease of negative symptoms. However, Andrade (2013) found that using a more neutral position for the anode, namely the left mastoid, caused the beneficial effects on AH to vanish. This implicates that the excitatory effect of the anode on the DLPFC is important to induce a beneficial effect on AH. Possibly this can be explained by a positive effect from anodal stimulation of the left DLPFC on working memory. This hypotheses is mainly derived from literature on tinnitus, for which tDCS has also been reported to be effective. Like schizophrenia, tinnitus has been associated with reduced executive control (Heeren et al., 2014). Besides, tinnitus has been found to be alleviated by tDCS over the DLPFC (Frank et al., 2012). These authors hypothesized that improved executive control, as induced by tDCS over the DLPFC, acts as a mediator for the impact from tDCS on tinnitus (Heeren et al., 2014). It may be that a similar therapeutic mechanism is involved in the therapeutic effect of tDCS for AH. Reduced inhibition of irrelevant verbal information may be an important underlying mechanism of AH, speculatively AH could result from misattribution of self-generated information to an external source (Daalman et al., 2011). Application of anodal tDCS over de left DLPFC has been found to enhance working memory, which plays an important role in the executive control of information (Brunoni and Vanderhasselt, 2014). It could therefore be speculated that anodal tDCS over the left DLPFC induces the ameliorating effects on AH by improving the inhibition of irrelevant verbal information. Whether this excitation must take place at the left DLPFC is, however, yet unclear, as the first case study in this field used the right supraorbital area for the anode and still showed a positive result (Homan et al., 2011). Moreover, tDCS does not only produce focal effects but also indirect effects on surrounding brain areas and their connections (Keeser et al., 2011). The more distal effects of tDCS could also be of influence on the ameliorating effects on AH. Imaging studies combined with tDCS in AH could provide more insight in the exact neurological underpinnings of the effects of this treatment.

Taken together, the results of tDCS as an alternative treatment option for AH are preliminary and so far mostly positive. However, a note of caution may be in place here. When new treatment strategies are introduced, the initial reports tend to feature relatively small sample sizes and favorable results, whereas small studies with negative findings do not tend to be published (Emerson et al., 2010; Sommer et al., 2012a). With an increase of studies with larger sample sizes over time, negative findings tend to become published as well. Such trends have led effect sizes to decrease per year of publication (Munafo and Flint, 2010). This effect has also been visible in the application of TMS for AH (Slotema et al., 2012). As tDCS in the treatment of AH is a young treatment method, future studies may well show less favorable results. In the University Medical Center Utrecht, we have started a large randomized double blind trial to investigate the effects of tDCS on AH. We plan to include 62 patients and expect to be able to report results in autumn 2015. It is important to await such studies with large sample sizes before conclusions are drawn about the efficacy of tDCS. This also means that there is a large responsibility for scientific periodic journals to also publish negative findings of such treatments, to help reduce publication bias for positive results.

Moreover, it is of importance that placebo treatments in double blind trials are hard to distinguish from active tDCS treatment. At this point, all placebo-controlled trials in this field have used tDCS at an intensity of 2 mA (Brunelin et al., 2012; Fitzgerald et al., 2014; Mondino et al., 2014). For 1 mA tDCS, it has been reported that placebo treatment is highly identical to active treatment. Both participants and investigators were not able to distinguish between the two (Gandiga et al., 2006). This provides confidence that both participants and investigators will remain well blinded throughout the duration of a double blind trial. However, it is yet unsure if the same applies for tDCS stimulation at 2 mA, as the physical sensations at this intensity are more pronounced and redness of the skin may occur. Although the literature is still unclear if participants themselves are able to differentiate between 2 mA active stimulation and placebo treatment, it has been found that redness of the skin is a possible clue for researchers to differentiate between the two (O’Connell et al., 2012; Palm et al., 2013). However, it is important that these results stem from crossover studies, in which subtle differences between treatments and the effects on each individual participant attract more attention. Brunoni et al. (2014) investigated tDCS blinding of 2 mA stimulation in a large sample of patients with a depression disorder, using a parallel design. Each patient received either active or placebo treatment. They found that patients were able to identify 2 mA active treatment above chance level, but this appeared to be related to the occurrence of clinical response. Patients who did not show clinical response to treatment were not able to identify active treatment. However, this study did not mention results about the accuracy of blinding in tDCS operators, which were different investigators than the assessors of the clinical effect of the treatment. Knowing if a parallel design has advantages for the accuracy of blinding in tDCS operators is important to assess the reliability of results from double blind studies using placebo-controlled tDCS in parallel designs.

At this time, studies using tDCS in AH have highly similar methods, using 15–20 min of treatment at 1–2 mA and mostly placing the electrodes over the left TPC and left DLPFC. Future research could further investigate if these are indeed the most optimal parameters and locations for tDCS in AH. In TMS, the left TPC is also the most used brain area for inhibitory stimulation as a treatment for AH. Multiple attempts at other locations for TMS did not lead to positive results compared to placebo (Slotema et al., 2014). Considering that, the choice for the left TPC as a location for the cathode in tDCS seems logical. However, it may be worth the effort to test the efficacy of both cathodal and anodal tDCS on other brain areas that have been associated with AH.

Furthermore, the precision of the treatment in influencing the targeted brain areas could possibly be optimized. In most studies, relatively large electrodes are used with a surface size of 35 cm^2^. However, it is also possible to use smaller electrodes with a surface size of 25 cm^2^ or even so called ‘high definition’ tDCS in which electrodes are used with a diameter of 12 mm or less (Minhas et al., 2010). Such a method may increase the precision of treatment by decreasing the amount of electricity that is lost due to distribution over the scalp. But using smaller electrodes should be investigated thoroughly, as the neuromodulating effects of small electrodes can strongly differ from those of big electrodes (Nitsche et al., 2008). It is possible that the desired effects are eliminated instead of increased using small electrodes, as they are designed for more focal effects and will have less effect on surrounding brain areas and their connections. The use of small electrodes also increases the need for more precision in placing the electrodes on the scalp. Currently, electrode placement is mostly done using the international 10–20 system for EEG electrode placement (Jasper, 1958), but it is unclear whether this offers enough precision when using smaller electrodes. A stereotactic neuronavigation technique based on an individual’s structural MRI image may be a suitable option to offer more reliability that the focus of tDCS treatment is targeted at the right brain area (Neggers et al., 2004; Fitzgerald et al., 2009; Sack et al., 2009).

## Conclusion

At this moment, tDCS appears to be a possible new treatment option to help reduce treatment-resistant AH. However, as currently only three randomized controlled trials have been published with relatively small sample sizes, two of which reporting positive results and one with non-significant results, insufficient data is available to determine if tDCS treatment is indeed effective in AH. Future placebo-controlled trials in large samples will have to assess if the mainly positive results up till now can be replicated. Furthermore, different methods and parameters for stimulation, localization, and precision of tDCS should be investigated in more detail to further optimize this treatment.
